# Scoring Single-Response Multiple-Choice Items: Scoping Review and Comparison of Different Scoring Methods

**DOI:** 10.2196/44084

**Published:** 2023-05-19

**Authors:** Amelie Friederike Kanzow, Dennis Schmidt, Philipp Kanzow

**Affiliations:** 1 Study Deanery University Medical Center Göttingen Göttingen Germany; 2 Department of Preventive Dentistry, Periodontology and Cariology University Medical Center Göttingen Göttingen Germany

**Keywords:** alternate-choice, best-answer, education, education system, educational assessment, educational measurement, examination, multiple choice, results, scoring, scoring system, single choice, single response, scoping review, test, testing, true/false, true-false, Type A

## Abstract

**Background:**

Single-choice items (eg, best-answer items, alternate-choice items, single true-false items) are 1 type of multiple-choice items and have been used in examinations for over 100 years. At the end of every examination, the examinees’ responses have to be analyzed and scored to derive information about examinees’ *true knowledge*.

**Objective:**

The aim of this paper is to compile scoring methods for individual single-choice items described in the literature. Furthermore, the metric *expected chance score* and the relation between examinees’ *true knowledge* and expected scoring results (averaged percentage score) are analyzed. Besides, implications for potential pass marks to be used in examinations to test examinees for a predefined level of *true knowledge* are derived.

**Methods:**

Scoring methods for individual single-choice items were extracted from various databases (ERIC, PsycInfo, Embase via Ovid, MEDLINE via PubMed) in September 2020. Eligible sources reported on scoring methods for individual single-choice items in written examinations including but not limited to medical education. Separately for items with n=2 answer options (eg, alternate-choice items, single true-false items) and best-answer items with n=5 answer options (eg, Type A items) and for each identified scoring method, the metric expected chance score and the expected scoring results as a function of examinees’ *true knowledge* using fictitious examinations with 100 single-choice items were calculated.

**Results:**

A total of 21 different scoring methods were identified from the 258 included sources, with varying consideration of correctly marked, omitted, and incorrectly marked items. Resulting credit varied between –3 and +1 credit points per item. For items with n=2 answer options, expected chance scores from random guessing ranged between –1 and +0.75 credit points. For items with n=5 answer options, expected chance scores ranged between –2.2 and +0.84 credit points. All scoring methods showed a linear relation between examinees’ *true knowledge* and the expected scoring results. Depending on the scoring method used, examination results differed considerably: Expected scoring results from examinees with 50% *true knowledge* ranged between 0.0% (95% CI 0% to 0%) and 87.5% (95% CI 81.0% to 94.0%) for items with n=2 and between –60.0% (95% CI –60% to –60%) and 92.0% (95% CI 86.7% to 97.3%) for items with n=5.

**Conclusions:**

In examinations with single-choice items, the scoring result is not always equivalent to examinees’ *true knowledge*. When interpreting examination scores and setting pass marks, the number of answer options per item must usually be taken into account in addition to the scoring method used.

## Introduction

**Figure 1:**
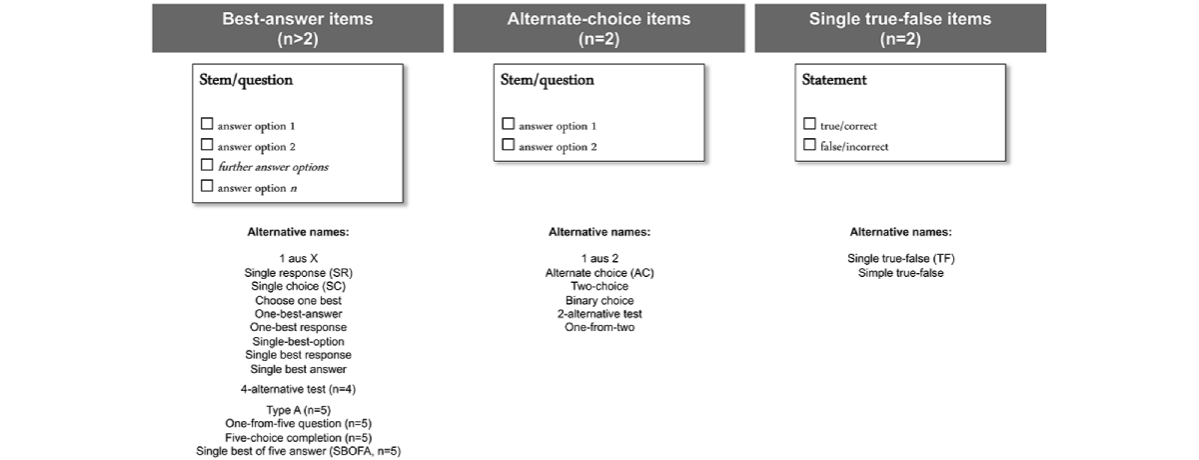
Examples of 3 different multiple-choice items in single-choice format and alternative designations used in the literature (no claim to completeness).

Multiple-choice items in single-response item formats (ie, single-choice items) require examinees to mark only 1 answer option or to make only 1 decision per item. The most frequently used item type among the group of single-choice items is the so-called best-answer items. Here, examinees must select exactly 1 (ie, the correct or most likely) answer option from the given answer options [1]. Often, best-answer items contain 5 answer options, although the number of answer options might vary (n≥2). Items with exactly 2 answer options are also referred to as alternative items (ie, alternate-choice items) [2]. In addition, single true-false items belong to the group of single-choice items. Examples of the mentioned single-choice items as well as alternative designations are shown in Figure 1.

Single-choice items have been used for more than 100 years to test examinees’ knowledge. The use of these items began among US school pupils, who were given alternate‑choice or best-answer items [3] or single true-false items [4] as a time-saving alternative to conventional open-ended questions (ie, essay-type examinations). Because of their character of only allowing clearly correct or incorrect responses from examinees, multiple-choice examinations were also called objective type examinations [5]. The term *new type examinations* was coined to distinguish them from previously commonly used open-ended questions [5,6].

The use of multiple-choice items did not remain exclusive to the setting of high schools but also extended to examinations in university contexts [7] and postgraduate medical education [8,9]. Today, multiple-choice items are frequently used in examinations of medical and dental students (eg, within the *United States Medical Licensing Examination*). Besides their usage in individual medical or dental programs, different multiple-choice item types found their way into examinations for medical students by the *National Board of Medical Examiners* [10]: within the context of single-choice items, those with n=5 were particularly used and referred to as Type A items.

Examinations aim at assessing examinees’ ability (ie, examinees’ *true knowledge* [k]) regarding predefined learning objectives. The downside when using multiple-choice examinations is that examinees might also mark an item correctly by guessing or by identifying the correct answer option through recognition. Thus, an active knowledge reproduction does not necessarily take place, and correct responses are not necessarily resulting from examinees’ *true knowledge*.

To grade examinees or to decide about passing or failing a summative examination based on a minimum required level of *true knowledge*, scoring algorithms are used to transfer examinees’ responses (ie, marking schemes) into a score. To assess examinees’ *true knowledge*, the obtained scores must either be reduced by the guessing factor, negative points (ie, malus points) must be assigned for incorrectly marked items, or the pass mark (ie, the corresponding cutoff score for the desired *true knowledge* cutoff value) must be adjusted based on the guessing probability [11]. The guessing probability for examinees without any knowledge (k=0, blind guessing only) amounts to 20% for single-choice items with n=5 and to 50% for alternate-choice items and single true-false items with n=2. Consequently, examinees without any knowledge score 20% or 50% of the maximum score on average, respectively [11]. However, it can be assumed that most examinees have at least partial knowledge (0<k<1) and that an informed guessing with remaining partial uncertainty occurs in most cases.

Since the introduction of multiple-choice items, numerous scoring methods have been described in the literature and (medical) educators are advised to choose an appropriate scoring method based on an informed decision. Therefore, the aim of this scoping review is (1) to map an overview of different scoring methods for individual single-choice items described in the literature, (2) to compare different scoring methods based on the metric *expected chance score*, and (3) to analyze the relation between examinees’ *true knowledge* and expected scoring results (averaged percentage score).

## Methods

### Systematic Literature Search

The literature search was performed according to the PRISMA-ScR (Preferred Reporting Items for Systematic reviews and Meta-Analyses extension for Scoping Reviews) checklist [12]. The checklist is available as Multimedia Appendix 1. As this review did not focus on health outcomes, the review was not registered at PROSPERO (International Prospective Register of Systematic Reviews) prior to its initiation.

### Eligibility Criteria

Potentially eligible sources were scientific articles, books, book chapters, dissertations, and congress abstracts reporting scoring methods for individual single-choice items in written examinations including but not limited to medical examinations. Scoring methods for item groups and scoring on examination level (eg, with different weighting of individual items, with mixed item types, or considering the total number of items per examination) were not assessed. Further, scoring methods that deviate from the usual marking procedure (ie, a single choice of marking exactly 1 answer option per item) were not considered. These include, for example, procedures that assess the confidence of examinees in their marking (eg, confidence weighting), let examinees select the incorrect answer options (eg, elimination scoring), let examinees narrow down the correct answer option (eg, subset selection), or allow for the correction of initially incorrectly marked items (eg, answer-until-correct). No further specifications were made regarding language, quality (eg, minimum impact factor), or time of publication.

### Information Sources

Four databases (ERIC, PsycInfo, Embase via Ovid, and MEDLINE via PubMed) were searched in September 2020. The search term was composed of various designations for single-choice items as well as keywords with regard to examinations. It was slightly adapted according to the specifications of the individual databases. The respective search terms for each database can be found in Table 1.

**Table 1:** Search terms used for each of the 4 databases.

Database	Search term
ERIC	(“single choice” OR “alternate choice” OR “single response” OR “one-best-answer” OR “single best response” OR “true-false” OR “Typ A”) AND (item OR items OR test OR tests OR testing OR score OR scoring OR examination OR examinations)
PsycInfo	(“single choice” OR “alternate choice” OR “single response” OR “one-best-answer” OR “single best response” OR “true-false” OR “Typ A”) AND (item OR items OR test OR tests OR testing OR score OR scoring OR examination OR examinations)
Embase via Ovid	((“single choice” or “alternate choice” or “single response” or “one-best-answer” or “single best response” or “true-false” or “Typ A”) and (item OR items or test or tests or testing or score or scoring or examination or examinations)).af.
MEDLINE via PubMed	(“single choice”[All Fields] OR “alternate choice”[All Fields] OR “single response”[All Fields] OR “one-best-answer” OR “single best response” OR “true-false”[All Fields] OR “Typ A”[All Fields]) AND (“item”[All Fields] OR “items”[All Fields] OR “test”[All Fields] OR “tests”[All Fields] OR “testing”[All Fields] OR “score”[All Fields] OR “scoring”[All Fields] OR “examination”[All Fields] OR “examinations”[All Fields])

### Selection of Sources

Literature screening, inclusion of sources, and data extraction were independently performed by 2 authors (AFK and PK). First, the titles and abstracts of the database results were screened. Duplicate results as well as records being irrelevant to the research question were sorted out. For books and book chapters, however, different editions were included separately. In a second step, full-texts sources were screened, and eligible records were included as sources. In addition, the references of included sources were searched in an additional hand search for further, potentially relevant sources. After each step, the results were compared, and any discrepancies were discussed until a consensus was reached. Information with regard to the described scoring methods was extracted using a piloted checklist.

### Data Extraction

The following data were extracted from included sources using a piloted spreadsheet if reported: (1) name of the scoring method, (2) associated item type, and (3) algorithm for calculating scores per item. The mathematical equations of each scoring method were adjusted to achieve normalization of scores up to a maximum of +1 point per item if necessary.

### Data Synthesis

For all identified scoring methods, the expected scoring results in case of pure guessing were calculated for single-choice items with n=2 and n=5 answer options, respectively [13]. The *expected chance score* is described in the literature as a comparative metric of different scoring methods [11,13-15]. For its calculation, examinees without any knowledge (k=0) are expected to always guess blindly and thus achieve the expected chance score on average.

In addition, expected scoring results for varying levels of k (0≤k≤1) were calculated. For examinees with partial knowledge (0<k<1), a correct response can be attributed to both partial knowledge and guessing, with the proportion of guessing decreasing as knowledge increases. By contrast, examinees with perfect knowledge (k=1) always select the correct answer option without the need for guessing [11].

Examinees were expected to answer all items, and it was supposed that examinees were unable to omit individual items or that examinees do not use an omit option. Furthermore, all items and answer options were assumed to be of equal difficulty and to not contain any cues. The calculation of the expected scoring result is shown in the following equation:



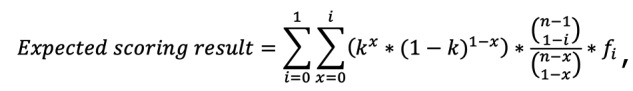



where f are the credit points awarded for a correctly marked item (i=1) or an incorrectly marked item (i=0) depending on the scoring method used; k is the examinees’ *true knowledge* [0≤k≤1]; n is the number of answer options per item; x=1 if the correct answer option is selected by *true knowledge*, otherwise x=0; in the equation shown, 0^0^ is defined as 1.

MATLAB software (version R2019b; The MathWorks) was used to calculate the relation between examinees’ *true knowledge* and the expected scoring results using fictitious examinations consisting of 100 single-choice items (all items with either n=2 or n=5).

## Results

### Overview

**Figure 2:**
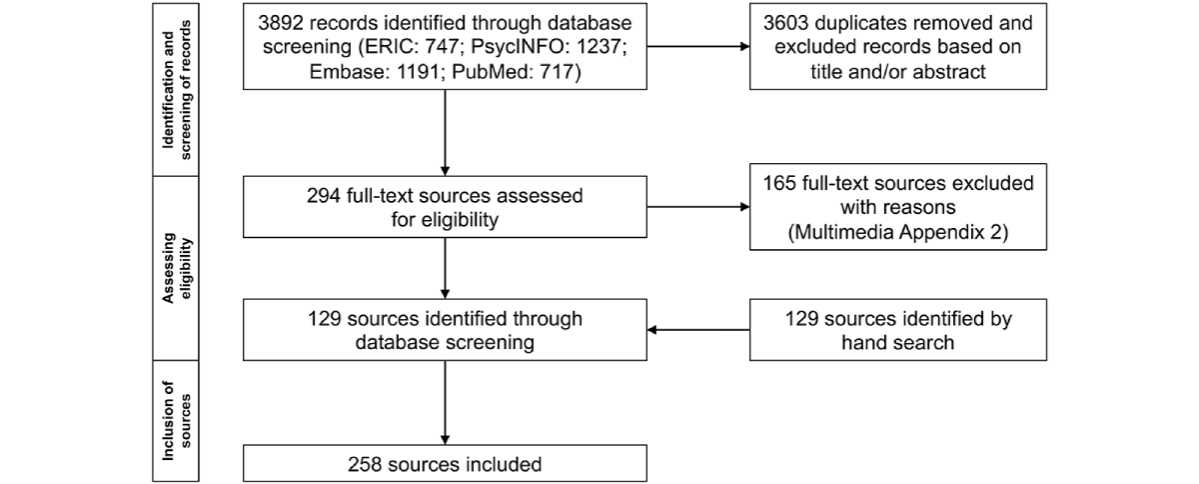
Flow diagram of systematic literature search.

Within the literature search, a total of 3892 records were found through database search. Of these, 129 sources could be included. A further 129 sources were identified from the references of the included sources by hand search. The entire process of screening and including sources is shown in Figure 2. Reasons for exclusion of sources during full-text screening are given in Multimedia Appendix 2.

The included sources describe 21 different scoring methods for single-choice items. In the following subsections, all scoring methods are described with their corresponding scoring formulas for calculating examination results as absolute scores (S). In addition, an overview with the respective scoring results for individual items as well as alternative names used in the literature is presented in Table 2. All abbreviations used throughout this review are listed at the end of this review.

**Table 2:** Identified scoring methods and algorithms for single-choice items.

Method number and sources	Scoring method	Algorithm^a-e^
1 [5,6,16-172]	0-1 score [167] Zero-one scoring [146] Binary scoring [146] Dichotomous scoring [105,114] All-or-none scoring [166] Number-right (NR) scoring [6,20,21,24,25,27,29-31,37,39,50,54,56,66,67,71,73,76,79,80,85,87,95,97,99,100,111,128,132,140,145,147,153,157,160,164] Number of right (NR) rule [139] No. right score (No Rt) [42] NC^f^ scoring [144] Rights score [72,82,92] R method [24,29,39] Number correct scoring [101,106,114,124,138,151,154,155] Percentage-correct scoring [165] Raw score [44-46,48,51,54,57,68,86,102,118,125,131,135] Score=rights [23,24] Uncorrected score [91,122,137] Conventional scoring [98] Rights-only score [62,87] 3 right minus 0 wrong [17]	f=1 (if i=1) f=0 (otherwise)
2 [37,41,46,53,58,60, 65,67,79-81,87,91, 98,111,122,137,173-180]	Formula scoring [67] Omission-formula scoring [79] Omit-correction [180] Positive scoring rule [139] Adjusted score [91]	f=1 (if i=1) f=1/n (if o=1) f=0 (otherwise)
3 [154]	Fair penalty [154]	f=1 (if i=1) f=0 (if o=1) f = 1 – 1/n (otherwise)
4 [181]	N/A^g^	f = 1/(n – 1) (if i=1) f=0 (if o=1) f=0 (otherwise)
5 [80,100,182]	N/A	f=1 (if i=1) f=0 (if o=1) f = –1/[2 (n – 1)] (otherwise)
6 [5,23-29,34,37,44, 46,48,50,51,53-57, 59-62,64,65,67,68, 70,71,74,75,79-81, 85-88,91,92,98-101, 105,106,111,113,120,122, 124-126,128,130,134,135, 137-139,144,145,160,169, 173-179,182-225]	Formula scoring [67,85,92,101,128,160,225] Conventional-formula scoring [79] Conventional correction-for-guessing formula [80,213] Conventional correction formula [201] “Neutral” counter-marking [88] CG^h^ scoring [144] Negative marking [130,145] Logical marking [130] Correction for blind guessing (CFBG) [135] Correction for guessing (CFG) formula [50,51,56,57,62,71,86,87,99,101,105,106,113,122,124,137,176,179,195,199,204,223] Correction for chance formula [56,87,174,188] Discouraging guessing [138] Rights minus wrongs correction [98] Corrected score [37,48,55,59,68,91] Classical score [207] Mixed rule [139]	f=1 (if i=1) f=0 (if o=1) f = –1/(n – 1) (otherwise)
7 [226]	N/A	f = 1/(n – 1) (if i=1) f=0 (if o=1) f = –1/(n – 1) (otherwise)
8 [41]	N/A	f = (n – 1)/n (if i=1) f=0 (if o=1) f = –1/n (otherwise)
9 [6,48,62,88,224,227,228]	3 right-wrong [6] Negative marking [228]	f=1 (if i=1) f=0 (if o=1) f=–1/3 (otherwise)
10^i^ [229]	N/A	f=1 (if i=1) f=0 (if o=1) f=–0.48 (otherwise)
11 [18,23,41,62,69,224,229-234]	N/A	f=1 (if i=1) f=0 (if o=1) f=–0.5 (otherwise)
12^i^ [229,231]	N/A	f=1 (if i=1) f=0 (if o=1) f=–0.6 (otherwise)
13 [4,6,16-19,21-25, 29-33,38,39,42,43, 45,49,52,55,69,72, 76,82,110,130,132,140, 143,154,157,164,172,190, 193,215,216,219,229,232, 233,235-267]	Formula scoring [157,164] Correct-minus-incorrect score [267] C-I score [132] R-W method [23,24,29,30,32,38,39,42,76,243,245,246,249,259] Number right minus number wrong method [39,45] Right-minus-wrong method [6,21,23,25,30,31,42,72,82,236,244,247] Rights minus wrongs method [29,253,254,256,258] Right-wrong [266] T-F formula [260] Guessing penalty [154] Correction-for-guessing [76,128] Negative marking [140] Logical marking [130] 1 right minus 1 wrong [17] Penal guessing formula [55] Corrected score [265]	f=1 (if i=1) f=0 (if o=1) f=–1 (otherwise)
14^i^ [249,268]	N/A	f=1 (if i=1) f=0.7 (if o=1) f=–1 (otherwise)
15^i^ [186]	N/A	f=1 (if i=1) f=0.7 (if o=1) f=–1.1 (otherwise)
16 [20]	N/A	f=1 (if i=1) f=0 (if o=1) f = –n/(n – 1) (otherwise)
17^i^ [203,259]	N/A	f=1 (if i=1) f=0 (if o=1) f=–1.5 (otherwise)
18^i^ [203]	N/A	f=1 (if i=1) f=0 (if o=1) f=–1.8 (otherwise)
19 [6,17,20,21,49,75,203,253,268-270]	Right – 2 wrong [6] 1 right minus 2 wrong [17] Rights minus two times wrongs [253] r-2w [253]	f=1 (if i=1) f=0 (if o=1) f = –2/(n – 1) (otherwise)
20^i^ [17,41]	1 right minus 3 wrong [17]	f=1 (if i=1) f=0 (if o=1) f=–3 (otherwise)
21^j^ [259]	N/A	f=1 (if i=1) f=0 (if o=1) f=–62/38 (if i=0 and t_m_=1) f=–38/62 (if i=0 and t_m_=0)

^a^f: resulting score per item.

^b^i=1 if the item was marked correctly; otherwise i=0.

^c^n: number of answer options per item (n≥2).

^d^o=1 if the item was omitted; otherwise o=0.

^e^t_m_=1 if the statement is true; otherwise t_m_=0.

^f^NC: number correct.

^g^N/A: not applicable (ie, no explicit name was previously introduced in literature).

^h^CG: correct for guessing.

^i^Only described for n=2.

^j^Only described for single true-false items.

### Scoring Methods Without Malus Points (0 to a Maximum of +1 Point per Item)

#### Method 1

One credit point is awarded for a correct response. Therefore, the examination result as absolute score (S) corresponds to the number of correct responses (R). No points are deducted for incorrect responses (W). The formula is S = R.

#### Method 2

One credit point is awarded for a correct response. In addition, 1/n credit points per item are awarded for each omitted item (O). No points are deducted for incorrect responses. The formula is S = R + O/n. This scoring method was first described by Lindquist [37] in 1951.

#### Method 3

One credit point is awarded for a correct response. For incorrect responses, 1 – 1/n credit points are awarded. The formula is S = R + (1 – 1/n)W. This scoring method was first described by Costagliola et al [154] in 2007 and named *fair penalty* by the authors. However, the term *penalty* is misleading because no points are deducted in case of incorrect responses.

#### Method 4

For each correct response, 1/(n – 1) credit points are awarded. Omitted items and incorrect responses do not affect the score. The formula is S = R/(n – 1). For example, 1 credit point is awarded for a correct response on single-choice items with n=2 (ie, alternate-choice items, single true-false items) but only 0.25 credit points are awarded for a correct response on best-answer items with n=5. This scoring method was first described by Foster and Ruch [181] in 1927.

### Scoring Methods With Malus Points (Maximum –1 to +1 Point per Item)

#### Method 5

One credit point is awarded for a correct response. For incorrect responses, 1/[2 (n – 1)] points are deducted. The formula is S = R – W/[2 (n – 1)]. This scoring method was first described by Little [182] in 1962.

#### Method 6

One credit point is awarded for a correct response. For incorrect responses, 1/(n – 1) points are deducted. The formula is S = R – W/(n – 1). This scoring method was first described by Holzinger [183] in 1924. For items with n=2, methods 6 and 13 result in identical scores; for items with n=4, methods 6 and 9 result in identical scores.

#### Method 7

For each correct response, 1/(n – 1) credit points are awarded. For an incorrect response, 1/(n – 1) points are deducted. The formula is S = (R – W)/(n – 1). This scoring method was first described by Petz [226] in 1978.

#### Method 8

For each correct response, (n – 1)/n credit points are awarded. For an incorrect response, 1/n points are deducted. Omissions do not affect the score. The formula is S = [(n – 1)/n]R – W/n. As a result, examinees achieve only 0.5 credit points for each correct response on single-choice items with n=2 and 0.8 credit points for each correct response on best-answer items with n=5. This scoring method was first described by Guilford [41] in 1954.

#### Method 9

One credit point is awarded for a correct response. For incorrect responses, 1/3 points are deducted. The formula is S = R – (1/3)W. Originally, this scoring method was described by Paterson and Langlie [6] in 1925 with the formula S = 3R – W for items with n=2 only. Later, the scoring method was also described for single-choice items with more answer options [88,203]. For items with n=4, methods 6 and 9 give identical results.

#### Method 10

One credit point is awarded for a correct response. For incorrect responses, 0.48 points are deducted. The formula is S = R – 0.48W. This scoring method was first described by Gupta and Penfold [229] in 1961 for single-choice items with n=2.

#### Method 11

One credit point is awarded for a correct response. Half a point is deducted for incorrect responses. The formula is S = R – 0.5 W. This scoring method was first described in 1924 by Brinkley [18] and Asker [230] for single-choice items with n=2, but was later also used for single-choice items with more answer options.

#### Method 12

One credit point is awarded for a correct response. For incorrect responses, 0.6 points are deducted. The formula is S = R – 0.6W. This scoring method was first described by Gupta [231] in 1957 for single-choice items with n=2.

#### Method 13

One credit point is awarded for a correct response. One point is deducted for incorrect responses. The formula is S = R – W. For items with n=2, methods 6 and 13 result in identical scores. This scoring method was first described by McCall [4] in 1920 for single-choice items with n=2, but was later also used for single-choice items with more answer options.

#### Method 14

This scoring method results in 1 credit point for a correct response, 0.7 credit points for an omitted item, and –1 point for an incorrect response. The formula is S = R + 0.7O – W. This scoring method was first described by Staffelbach [268] in 1930 for single-choice items with n=2.

### Scoring Methods With Malus Points (Maximum –3 to +1 Points per Item)

#### Method 15

This scoring method results in 1 credit point for a correct response, 0.7 credit points for an omitted item, and –1.1 points for an incorrect response. The formula is S = R + 0.7O – 1.1W. This scoring method was first described by Kinney and Eurich [186] in 1933 for items with n=2.

#### Method 16

One credit point is awarded for a correct response. For an incorrect response, n/(n – 1) points are deducted. The formula is S = R – nW/(n – 1). This scoring method was first described by Miller [20] in 1925. For items with n=2, methods 16 and 19 result in identical scores.

#### Method 17

For an incorrect response, 1.5 times as many points are deducted as credit points are awarded for a correct response. The original scoring formula is S = 2R – 3W. If a maximum of 1 credit point is awarded per item, 1 credit point is awarded for a correct response and 1.5 points are deducted for an incorrect response. This results in the following scoring formula: S = R – 1.5W. This scoring method was first described by Cronbach [259] in 1942 for items with n=2.

#### Method 18

One credit point is awarded for a correct response. For an incorrect response, 1.8 points are deducted. The scoring formula is S = R – 1.8W. This scoring method was first described by Lennox [203] in 1967 for items with n=2.

#### Method 19

One credit point is awarded for a correct response. For an incorrect response, 2/(n – 1) points are deducted. The formula is S = R – 2W/(n – 1). This scoring method was first described by Gates [269] in 1921 with the scoring formula S = R – 2W for items with n=2. Later, the scoring formula was also described for single-choice items [203,270]. In case of items with n=2, methods 16 and 19 result in identical scores.

#### Method 20

One credit point is awarded for a correct response. Three points are deducted for an incorrect response. The formula is S = R – 3W. This method was first described by Wood [17] in 1923 for items with n=2.

### Specific Scoring Methods for Single True-False Items

#### Method 21

One credit point is awarded for correctly identifying the statement of true-false single items as true or false. If the statement presented is marked incorrectly, 62/38 points are deducted on true statements (W_t_, incorrectly marked as false), but only 38/62 points are deducted on false statements (W_f_, incorrectly marked as true). The scoring formula is S = R – (62/38)W_t_ – (38/62)W_f_. This scoring method was first described by Cronbach [259] in 1942 for single true-false items and differentiates in the scoring of incorrectly marked true/false statements.

### Expected Chance Scores of the Identified Scoring Methods

The expected chance scores of examinees without any knowledge (k=0) vary between –1 and +0.75 credit points per item for single-choice items with n=2. For single-choice items with n=5, expected chance scores show a larger variability. Here, the expected chance scores vary between –2.2 and +0.84 credit points per item, depending on the selected scoring method. A detailed list is shown in Table 3.

**Table 3:** Overview of scoring results for single-choice items with either n=2 or n=5 answer option.

Method number	Scoring formula^a-f^	n^g^=2				n=5		
		Credit for incorrect responses^h^	Credit for correct responses^i^	Expected chance score	Credit for incorrect responses^h^		Credit for correct responses^i^	Expected chance score
1	S = R	0	1	0.50	0		1	0.20
2	S = R + O/n	0	1	0.50	0		1	0.20
3	S = R + (1 – 1/n)W	0.50	1	0.75	0.80		1	0.84
4	S = R/(n – 1)	0	1	0.50	0		0.25	0.05
5	S = R – W/[2 (n – 1)]	–0.50	1	0.25	–1/8		1	0.10
6	S = R – W/(n – 1)	–1	1	0.00	–0.25		1	0.00
7	S = (R – W)/(n – 1)	–1	1	0.00	–0.25		0.25	0.15
8	S = [(n – 1)/n]R – W/n	–0.50	0.50	0.00	–0.20		0.80	0.00
9	S = R – (1/3)W	–1/3	1	1/3	–1/3		1	–2/30
10	S = R – 0.48W	–0.48	1	0.26	–0.48		1	–23/125
11	S = R – 0.5W	–0.50	1	0.25	–0.5		1	–0.20
12	S = R – 0.6W	–0.60	1	0.20	–0.6		1	–0.28
13	S = R – W	–1	1	0.00	–1		1	–0.60
14	S = R + 0.7O – W	–1	1	0.00	–1		1	–0.60
15	S = R + 0.7O – 1.1W	–1.10	1	–0.05	–1.10		1	–0.68
16	S = R – nW/(n – 1)	–2	1	–0.50	–1.25		1	–0.80
17	S = R – 1.5W	–1.50	1	–0.25	–1.5		1	–1.00
18	S = R – 1.8W	–1.80	1	–0.40	–1.8		1	–1.24
19	S = R – 2 W/(n – 1)	–2	1	–0.50	–0.5		1	–0.20
20	S = R – 3W	–3	1	–1.00	–3		1	–2.20
21	S = R – (62/38)W_t_ – (38/62)W_f_	–62/38 or –38/62	1	N/A^j^	–62/38 or –38/62		1	N/A^j^

^a^S: examination result as absolute score.

^b^R: number of correct responses.

^c^O: number of omitted items.

^d^W: number of incorrect responses.

^e^W_t_: number of true statements incorrectly marked as false.

^f^W_f_: number of false statements incorrectly marked as true.

^g^n: number of answer options per item.

^h^R=0, O=0, W=1.

^i^R=1, O=0, W=0.

^j^Expected chance scores were not calculated for method 21, because these depend on the proportion of true-false items with correct or incorrect statements.

### Relation Between Examinees’ *true knowledge* and the Expected Scoring Results

**Figure 3:**
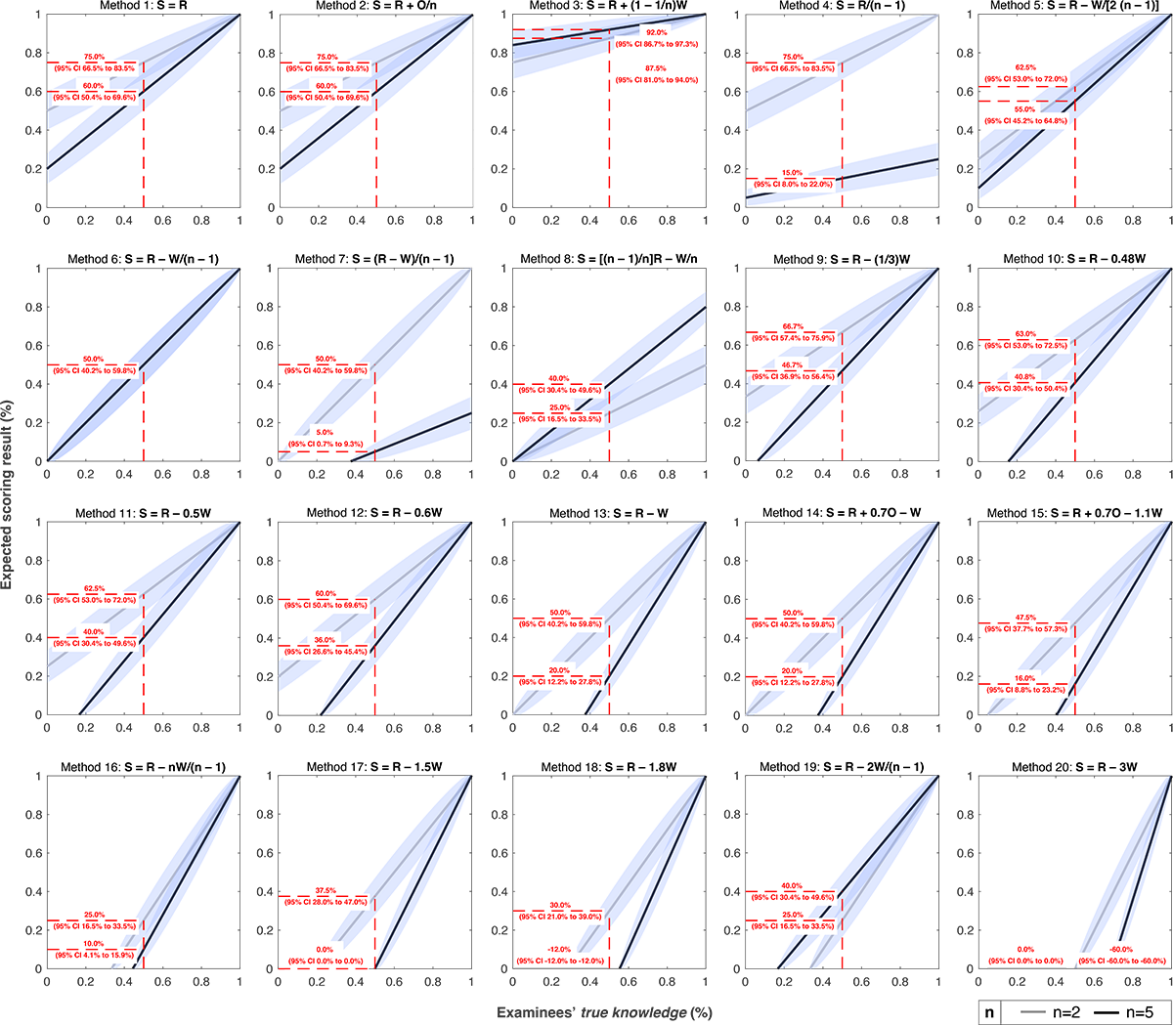
Relation between examinees’ *true knowledge* (%) and the expected scoring results for examinations with 100 single-choice items (either n=2 or n=5 answer options per item). In each case, the expected scoring result at 50% *true knowledge* is shown with the associated 95% confidence interval. Method 21 is not shown because the relation depends on the proportion of single true-false items with true or false statements. O: number of omitted items (O=0); R: number of correct responses; S: examination result as absolute score (max. up to 100 points); W: number of incorrect responses.

The relation between examinees’ *true knowledge* and expected scoring results for single-choice items with n=2 and n=5 is shown in Figure 3. For all identified scoring methods, there is a linear relation between examinees’ *true knowledge* and the expected scoring results. However, some scoring methods (ie, methods 4 and 7) award less than 1 point for correctly marked items if there are more than 2 answer options (n>2). One further method (method 8) awards less than 1 point for correctly marked items regardless of the number of answer options, so the maximum score for these scoring methods might be less than 100%. Depending on the scoring method and the number of answer options, the y-axis intercepts (expected chance scores, k=0) and the slopes differ. A low expected chance score results in a wide range of examination results that differentiate different examinees’ knowledge levels (ranging from the expected chance score as the lower limit to the maximum score as the upper limit). Only for methods 6 and 8 as well as method 7 in the case of n=2, the line starts from the pole (ie, examinees without any knowledge [k=0] achieve an examination result of 0%). Only for method 6, the relation between examinees’ *true knowledge* and the expected scoring results is independent of the number of answer options per item.

## Discussion

### Principal Findings

In this review, a total of 21 scoring methods for single-choice items could be identified. The majority of identified scoring methods is based on theoretical considerations or empirical findings, while others have been arbitrarily determined. Although some methods were only described for certain item types (ie, single-choice items with n=2), most of them might also be used for scoring items with more answer options. However, 1 method is suitable for scoring single true-false items only.

All scoring methods have in common that omitted items do not result in any credit deduction. Some scoring methods even award a fixed amount of 0.7 points on omitted items (methods 14 and 15), which is, however, lower than the full credit for a correct response, or the score to be achieved on average by guessing (1/n, method 2).

For the identified scoring methods, the possible scores range from a maximum of –3 to +1 points. A correctly marked item is usually scored with 1 full point (1 credit point). Exceptions to this are 3 scoring methods that only award 1 credit point in case of single-choice items with n=2 (methods 4 and 7) or that never award 1 credit point (method 8). These scoring methods are questionable because as the number of answer options increases, the guessing probability decreases. Further, a differentiation between examinees’ marking on true and false statements (method 21) is not justified, because the importance of correctly identifying true statements (ie, correctly marking the statement as true) and false statements (ie, correctly marking the statement as false) is likely to be considered equivalent in the context of many examinations.

With the exception of method 6, the relation between examinees’ *true knowledge* and the resulting examination scores depends on the number of answer options per item (n). Therefore, the number of answer options per item must usually be taken into account when examination scores are interpreted.

Examinations are designed to determine examinees’ knowledge as well as to decide whether the examinees pass or fail in summative examinations. It can be generally assumed that examinees must perform at least 50% of the expected performance to receive at least a passing grade [271]. If examinees are to be tested on a *true knowledge* of 50%, adjusted pass marks must be applied depending on the scoring method used and the number of answer options per item. The theoretical considerations show that for an examination testing for 50% **true knowledge*,* a pass mark of 0% or even negative scoring results might be appropriate, while other scoring methods would require pass marks up to 92%. Consequently, the examination’s pass mark must be considered or adjusted when selecting a suitable scoring method. However, the pass mark might be fixed due to local university or national guidelines resulting in a limited number of suitable scoring methods.

### Correction for Guessing

To account for guessing in case of single true-false items, the scoring formula R – W (method 13) was originally propagated in the literature, where the number of incorrect responses is subtracted from the number of correct responses [4]. Since its first publication in 1920, this scoring method has been frequently criticized: the main criticism is that this scoring method assumes examinees to either have complete knowledge (k=1) or to guess blindly (k=0). However, especially in the context of university examinations, examinees are assumed to have at least some partial knowledge. Furthermore, the scoring method assumes that incorrect responses are exclusively the result of guessing. No differentiation is made between incorrect responses due to blind guessing (ie, complete lack of knowledge), informed guessing (ie, guessing with partial knowledge and remaining uncertainty), or other reasons (eg, transcription errors introduced when transferring markings to the answer sheet) despite complete knowledge. Because of the 50% guessing probability in case of alternate-choice items or single true-false items, it is assumed that for each incorrectly guessed response (W) 1 item is also marked correctly by guessing on average, so that the corrected result is obtained by the scoring formula R – W. Especially in the case of partial knowledge, examinees’ marking behavior not only depends on their actual knowledge but also on their individual personality (eg, risk-seeking behavior) [272]. Consequently, the construct validity of examinations must be questioned when using the scoring formula R – W. Another criticism is that a correction by awarding malus points does not change the relative ranking of the results of different examinees if all examinees have sufficient time to take the examination and all items are answered [44,46].

Therefore, alternative scoring methods and scoring formulas emerged in addition to the already discussed scoring formula R – W. In this context, the literature often refers to formula scoring. However, the term *formula scoring* is not used uniformly: on the one hand, it is used as a general umbrella term for various scoring methods to correct for the guessing probability. On the other hand, the term is used to refer to specific scoring methods (methods 2, 6, and 13). Using method 2, examinees receive 1/n points for each omitted item. This corresponds to the number of points they would have scored on average by blindly guessing. Method 6 is a generalization of the scoring formula R – W for variable numbers of answer options. In case of n answer options, there are n – 1 times as many incorrect answer options as correct answer options and it is assumed that for each incorrectly guessed response (W) also W/(n – 1) items are marked correctly by guessing on average. Therefore, the corrected score is given by the scoring formula R – W/(n – 1). Consequently, methods 6 and 13 yield identical scoring results in case of items with n=2.

### Strengths and Limitations

So far, the relation between examinees’ *true knowledge* and the expected scoring result for single-choice items has been shown only for a small number of scoring methods [273]. Therefore, a systematic literature search was conducted in several databases as part of this review. As a result, a large number of different scoring methods have been identified and were compared in this review assisting (medical) educators in gaining a comprehensive overview and to allow for informed decisions regarding the scoring of single-choice items. However, limitations are also present: First, a number of assumptions (eg, equal difficulty of items and answer options, absence of cues) were required for simplification of the calculations and comparisons. However, these assumptions are likely to be violated in real examinations [15,274-276]. Second, calculations are based on classical test theory assumptions and did not employ item response theory models that might yield different results. Third, databases were already searched in September 2020 and potentially eligible sources published thereafter might not be included in this review. However, single-choice items have been used in examinations for over 100 years and further scoring methods are unlikely to have emerged in the past 2 years.

### Comparison With Prior Work

Although some of the identified scoring methods might also be applied to other item formats (eg, *multiple-select items*), the presented equation for the calculation of the expected scoring result is limited to single-choice items. Analogous calculations for items in multiple-select multiple-choice formats with (eg, Pick-N items) or without (eg, Multiple-True-False items) mutual stochastic dependence have already been described in the literature [11,14].

### Practical Implications

In practice, the evaluation of a multiple-choice examination should be based on an easy-to-calculate scoring method that allows for a transparent credit awarding and is accepted by jurisdiction. In this regard, scoring methods with malus points (ie, methods 5-21) may not be accepted by national jurisdiction in certain countries (eg, Germany) [277]. Furthermore, it does not seem reasonable to discourage examinees from marking an item by awarding malus points for the reasons already mentioned. Therefore, only 4 of the presented scoring methods can be versatilely used. Furthermore, it seems inconclusive to reward partial credit on incorrect responses or to refrain from awarding 1 credit point for correct responses in case of items with more than 2 answer options (n>2). As a result, only a dichotomous scoring method (1 credit point for a correct response, 0 points for an incorrect response or omitted items) is recommended. Within the context of this review, the outlined scoring method is referred to as method 1.

The scoring of examinations with different item types, item formats, or items containing a varying number of answer options within a single examination is more complicated. Here, the individual examination sections would have to be evaluated separately or the credit resulting from the respective item type or item format would have to be corrected to enable a uniform pass mark. For example, in the single-choice format, credit points resulting from items with n=2 would have to be reduced to compensate for the higher guessing probability compared with items with n=5 (ie, 50% vs 20% guessing probability).

### Conclusions

Single-response items only allow clearly correct or incorrect responses from examinees. Consequently, the scoring should also be dichotomous and result in either 0 points (incorrect response) or 1 credit point (correct response) per item. Because of the possibility of guessing, scoring results cannot be equated with examinees’ *true knowledge*. If (medical) educators interpret scoring results and determine suitable pass marks, the expected chance score must be taken into account, which in the proposed dichotomous scoring methods depends on the number of answer options per item.

## Appendix

Multimedia Appendix 1 PRISMA-ScR (Preferred Reporting Items for Systematic reviews and Meta-Analyses extension for Scoping Reviews) checklist.

Multimedia Appendix 2 Excluded sources after screening of full texts.

## Abbreviations

CG: correct for guessing
f: resulting score per item
k: examinees’ *true knowledge*
n: number of answer options per item
NC: number correct
O: number of omitted items
PRISMA-ScR: Preferred Reporting Items for Systematic reviews and Meta-Analyses extension for Scoping Reviews
PROSPERO: International Prospective Register of Systematic Reviews
R: number of correct responses
S: examination result as absolute score
W: number of incorrect responses
W_f_: number of false statements incorrectly marked as true
W_t_: number of true statements incorrectly marked as false

## Data Availability

All data generated during or analyzed during this study are included in this published article and its supplementary information files.
