# Supplementary material for: Scoring Single-Response Multiple-Choice Items: Scoping Review and Comparison of Different Scoring Methods
Source: JMIR Med Educ. 2023 May 19;9:e44084. doi: 10.2196/44084 (PMC10238964; doi:10.2196/44084)
Supplement: Multimedia Appendix 2 [file mededu_v9i1e44084_app2.docx]

**Multimedia Appendix 2**

*Excluded sources after screening of full-texts*

| Reason for exclusion | Source |
| --- | --- |
| No relevant information related to specific scoring algorithms | Brabec et al. 2020 [1]; Wen et al. 2020 [2]; Brassil und Couch 2019 [3]; Rahmawati 2019 et al. [4]; Santana-Vaz 2018 [5]; Seoana 2018 [6]; Tourangeau et al. 2016 [7]; Apple 2015 [8];Brenner et al. 2015 [9]; Chagín et al. 2015 [10]; Lindner et al. 2015 [11]; Nguyen et al. 2015 [12]; Tetteh und Sarpong 2015 [13]; Schaap et al. 2014 [14]; Sharma und Mutalik 2014 [15]; McAllister und Guidice 2012 [16]; Chandratilake et al. 2011 [17]; Foster 2011 [18]; Salend 2011 [19]; Abid et al. 2010 [20]; Kwan 2010 [21]; Adebule 2009 [22]; Kelly und Dennick 2009 [23]; Khare und Lam 2008 [24]; McCoubrie und McKnigh 2008 [25]; Paulino und Kurtz 2008 [26]; Tan und McAleer 2008 [27]; Vyas und Supe 2008 [28]; Dunham 2006 [29]; Al-Rubaish et al. 2005 [30]; Dudley 2004 [31]; Shojima und Toyoda 2004 [32]; Downing 2003 [33]; Rahim und Abumadini 2003 [34]; Zimmermann und Williams 2003 [35]; Conderman und Koroghlanian 2002 [36]; Ercikan 2002 [37]; Rieck 2002 [38]; Vacc et al. 2001 [39]; Fowell et al. 2000 [40]; Dodds 1999 [41]; Herskovic 1999 [42]; Oosterhof 1999 [43]; Parsons und Fenwick 1999 [44]; Tomey 1999 [45]; Johanson und Motlomelo 1998 [46]; Natal 1998 [47]; Sahai et al. 1998 [48]; Fenderson et al. 1997 [49]; Wang und Calhoun 1997 [50]; Gose und Escudero 1996 [51]; Rogers 1996 [52]; Smith 1995 [53]; Wang 1995 [54]; Graham and Isom 1994 [55]; Halpin et al. 1994 [56]; Pokrajac und Čulo 1994 [57]; Hancock et al. 1993 [58]; Toppino und Luipersbeck 1993 [59]; Dowd 1992 [60]; Downing 1992 [61]; Frisbie 1992 [62]; Haladyna 1992 [63]; Pinglia 1992 [64]; Williams 1992 [65]; Van Susteren et al. 1991 [66]; Frisbie und Becker 1990 [67]; Powell und Gillespie 1990 [68]; Rubadeau et al. 1990 [69]; Holden 1989 [70]; Kolstad und Kolstad 1989 [71]; Norcini et al. 1989 [72]; Petz 1989 [73]; Shick 1989 [74]; Tppino und Brochin 1989 [75]; Johanson und Motlomelo 1988 [46]; Kolstad et al. 1987 [76]; Luecht 1987 [77]; Maihoff 1987 [78]; Smith 1987 [79]; Cirn 1986 [80]; Frisbie und Druva 1986 [81]; Van Susteren 1986 [82]; Vydareny et al. 1986 [83]; Haladyna und Downing 1985 [84]; Kolstad et al. 1985 [85]; Maihoff und Mehrens 1985 [86]; Petz 1985 [87]; Varnhagen und Calder 1985 [88]; Dolinsky, Reid 1984 [89]; Georgi 1984 [90]; Ory 1983 [91]; Barker und Ebel 1982 [92]; Koeslag 1982 [93]; McKee und Lang 1982 [94]; Weiten 1982 [95]; Hogan 1981 [96]; Koeslag und Melzer 1981 [97]; Ebel 1980 [98]; Hsu 1980 [99]; Sax und Reiter 1980 [100]; Green 1979 [101]; Hsu 1979 [102]; Ebel 1978 [103]; Green 1978 [104]; Knapp 1977 [105]; Larkins und Swint 1976 [106]; Ebel 1975 [107];Boyd 1974 [108]; Follmann et al. 1974 [109]; Frisbie 1974 [110]; Olsen und Barickowski 1974 [111]; Sheehan und Hambleton 1974 [112]; Ebel 1973 [113]; Frisbie 1973 [114]; Frisbie und Ebel 1972 [115]; Knight 1972 [116]; Lynch 1972 [117]; Oosterhof und Glasnapp 1972 [118]; Ebel 1971 [119]; Ebel 1971 [120]; Frisbie 1971 [121]; Jackson et al. 1971 [122]; Ebel 1970 [123]; van der Veen et al. 1970 [124]; Van Naerssen 1970 [125]; Ebel 1968 [126]; Avital 1967 [127]; Zern 1967 [128]; Gustav 1964 [129]; Robinson 1957 [130]; Carter 1955 [131]; Travers 1950 [132]; Weimer 1949 [133]; Wright 1944 [134]; Copeland und Gilliland 1943 [135]; Curtis et al. 1943 [136]; Carter und Crone 1940 [137]; Drake 1940 [138]; Andrew und Bird 1938 [139]; Dunlap 1938 [140]; Bird und Andrew 1937 [141]; Buckingham 1936 [142]; Hovland und Eberhart 1935 [143]; Fay und Middleton 1934 [144]; Jordan 1934 [145]; Lee und Symonds 1934 [146]; Noll 1934 [147]; Krueger 1933 [148]; Eurich 1931 [149]; Cuff 1932 [150]; Shulson und Crawford 1928 [151]; Fritz 1927 [152]; Ruch und Stoddard 1927 [153]; McClusky und Curtis 1926 [154]; Ruch et al. 1926 [155]; Wood 1926 [156]; Wood 1926 [157]; Remmers et al. 1923 [158]; Knight 1922 [159] |
| Scoring algorithm allows examinees to correct initially incorrect responses | Irvin et al. 1980 [160] |
| Scoring algorithm awards credit at the level of complete examinations | Tarnóczy 1938 [161] |
| Scoring algorithm is designed for Multiple True False items | Dudley 2006 [162]; Sim und Rasiah 2006 [163]; Albanese et al. 1979 [164]; Mayo und Siegel 1956 [165] |

**References for Multimedia Appendix 2**

1. Brabec JA, Pan SC, Bjork EL, Bjork RA. True-false testing on trial: guilty as charged or falsely accused? Educ Psychol Rev 2020;33(2):667-92.

2. Wen J, Lu W, Chen Z. Innovation and construction of examination database of Pharmacology. Indian J Pharm Educ Res 2020;54(2):279-83.

3. Brassil CE, Couch BA. Multiple-true-false questions reveal more thoroughly the complexity of student thinking than multiple-choice questions: a Bayesian item response model comparison. Int J STEM Educ 2019;6:16.

4. Rahmawati LE, Suwandi S, Saddhono K, Setiawan B. Construction of test instrument to assess foreign student’s competence of Indonesian language through objective test. Int J Instr 2019;12(4):35-48.

5. Santana-Vaz N, Pairaudeau C, Burri N, Amarasekara A. The educated and wild guess within postgraduate anaesthetic examinations: Can a purposefully developed guessing guide improve multiple true false and single best answer question scores? Anaesthesia 2018;73(S3):114.

6. Seoane JJG. The impact of multiple choice questions in Physiology learning. How to write multiple choice questions in Physiology. J Physiol Biochem 2018;74(S1):S11.

7. Tourangeau R, Maitland A, Yan HY. Assessing the scientific knowledge of the general public. Public Opin Q 2016;80(3):741-60.

8. Apple KJ. Evaluating learning: Designing effective quizzes, tests, and final exams for Psychology courses. In: Dunn DS, editor. The Oxford handbook of undergraduate psychology education. Oxford, England: Oxford University Press; 2015.

9. Brenner E, Chirculescu ARM, Reblet C, Smith C. Assessment in anatomy. Eur J Anat 2015;19(1):105-24.

10. Chagín MCA, Carreño EJV, Rodríguez CJR. Falso o verdadero, ¿es esa la pregunta? [True or false: Is that the question?]. Iatreia 2015;28(2):120-7.

11. Lindner MA, Strobel B, Köller O. Multiple-Choice-Prüfungen an Hochschulen? Ein Literaturüberblick und Plädoyer für mehr praxisorientierte Forschung [Are multiple-choice exams useful for universities? A literature review and argument for a more practice oriented research]. Z Pädagog Psychol 2015;29(3-4):133-49.

12. Nguyen DQ, Patenaude JV, Gagnon R, Deligne B, Bouthillier I. Simulation-based multiple-choice test assessment of clinical competence for large groups of medical students: a comparison of auscultation sound identification either with or without clinical context. Can Med Educ J 2015;6(1):e4-e13.

13. Tetteh GA, Sarpong FA-A. Influence of type of assessment and stress on the learning outcome. J Int Educ Bus 2015;8(2):125-44.

14. Schaap L, Verkoeijen P, Schmidt H. Effects of different types of true–false questions on memory awareness and long-term retention. Assess Eval High Educ 2014;39(5):625-40.

15. Sharma HS, Mutalik MM. Comparison between student scores in multiple choice questions and structured essay questions at III MBBS examination in Otorhinolaryngology in a medical college in Mauritius. Int J Pharm Bio Sci 2014;5(4):1195-202.

16. McAllister D, Guidice RM. This is only a test: A machine-graded improvement to the multiple-choice and true-false examination. Teach High Educ 2012;17(2):193-207.

17. Chandratilake M, Davis M, Ponnamperuma G. Assessment of medical knowledge: The pros and cons of using true/false multiple choice questions. Natl Med J India 2011;24(4):225-8.

18. Foster N. Analysis of short-answer question styles versus gender in pre-clinical veterinary education. J Vet Med Educ 2011;38(1):67-73.

19. Salend SJ. Creating student-friendly tests. Educ Leadersh 2011;69(3):52-8.

20. Abid K, Qureshi AL, Yasmin R. Continuum of medical education: objectivity in instructional method, learning and assessment. J Pak Med Assoc 2010;60(4):262-4.

21. Kwan FB. True/false test: enhancing its power through writing. J Instruct Pedagog 2010;4.

22. Adebule SO. Reliability and levels of difficulty of objective test items in a Mathematics achievement test: a study of ten senior secondary schools in five local government areas of Akure, Ondo State. Educ Res Rev 2009;4(11):585-7.

23. Kelly S, Dennick R. Evidence of gender bias in true-false-abstain medical examinations. BMC Med Educ 2009;9:Article 32. PMID:19500414

24. Khare A, Lam H. Assessing student achievement and progress with online examinations: some pedagogical and technical issues. Int J E-Learn 2008;7(3):383-402.

25. McCoubrie P, McKnight L. Single best answer MCQs: a new format for the FRCR part 2a exam. Clin Radiol 2008;63(5):506-10.

26. Paulino AC, Kurtz E. American College of Radiology in-training examination for residents in radiation oncology (2004-2007). Int J Radiat Oncol Biol Phys 2008;70(3):666-70.

27. Tan LT, McAleer JJ, Final FRCR Examination Board. The introduction of single best answer questions as a test of knowledge in the final examination for the fellowship of the Royal College of Radiologists in Clinical Oncology. Clin Oncol (R Coll Radiol) 2008;20(8):571-6.

28. Vyas R, Supe A. Multiple choice questions: a literature review on the optimal number of options. Natl Med J India 2008;21(3):130-3.

29. Dunham ML. An investigation of the multiple true-false item for nursing licensure and potential sources of construct-irrelevant difficulty. Lawrence, KS: University of Kansas; 2006.

30. Al-Rubaish AM, Al-Umran KU, Wosornu L. An audit of assessment tools in a medical school in eastern Saudi Arabia. J Family Community Med 2005;12(2):101-5.

31. Dudley AP. The viability of the multiple true-false test format in second language testing. Philadelphia, PA: Temple University; 2004.

32. Shojima K, Toyoda H. Item parameter estimation when a test contains different item response models. Jap J Educ Psychol 2004;52(1):61-70.

33. Downing SM. Guessing on selected-response examinations. Med Educ 2003;37(8):670-1.

34. Rahim SI, Abumadini MS. Comparative evaluation of multiple choice question formats. Introducing a knowledge score. Neurosciences (Riyadh) 2003;8(3):156-60. PMID:23649110

35. Zimmerman DW, Williams RH. A new look at the influence of guessing on the reliability of multiple-choice tests. Appl Psychol Meas 2003;27(5):357-71.

36. Conderman G, Koroghlanian C. Writing test questions like a pro. Interv Sch Clin 2002;38(2):83-7.

37. Ercikan K. Scoring examinee responses for multiple inferences: Multiple scoring in assessments. Educ Meas 2002;21(2):8-14.

38. Rieck WA. Putting assessment to the test: tips for designing standards-based tests. Sci Teach 2002;69:46-9.

39. Vacc NA, Loesch LC, Lubik RE. Writing Multiple-Choice Test Items. In: Walz GR, Bleuer JC, editors. Assessment: Issues and Challenges for the Millennium. Greensboro, NC: CAPS Publications; 2001.

40. Fowell SL, Maudsley G, Maguire P, Leinster SJ, Bligh J. Student assessment in undergraduate medical education in the United Kingdom, 1998. Med Educ 2000;34(S1):1-49.

41. Dodds J. Writing good tests for student grading or research purpose: some basic precepts and principles. Annual Meeting of the Southwest Educational Research Association; San Antonio, TX; 1990.

42. Herskovic P. Reutilization of multiple-choice questions. Med Teach 1999;21(4):430-1.

43. Oosterhof A. Developing and Using Classroom Assessments. 2^nd^ ed. Upper Saddle River, NJ: Prentice Hall; 1999.

44. Parsons J, Fenwick T. Using objective tests to evaluate. Edmonton, Alberta, Canada; 1999.

45. Tomey AM. Selected response test items. Nurse Educ 1999;24(5):9-13.

46. Johanson G, Motlomelo S. An item format continuum for classroom assessment. Annual Meeting of the American Educational Research Association; San Diego, CA; 1988.

47. Natal D. On-line assessment: what, why, how. Technology Education Conference; Santa Clara, CA; 1998.

48. Sahai V, Demeyere P, Poirier S, Píro F. Measuring the consistency in change of Hepatitis B knowledge among three different types of tests: True/false, multiple choice, and fill in the blanks tests. Can J Program Eval 1998;13(2):123-8.

49. Fenderson BA, Damjanov I, Robeson MR, Veloski JJ, Rubin E. The virtues of extended matching and uncued tests as alternatives to multiple choice questions. Hum Pathol 1997;28(5):526-32.

50. Wang J, Calhoun G. A useful function for assessing the effect of guessing on true-false and multiple-choice tests. Educ Psychol Meas 1997;57(1):179-85.

51. Gose MD, Escudero RM. Whether to use true-false test items. Educ Res Quart 1996;20(1):37-47.

52. Rogers JB. Single-response and multiple-choice response formats on a sixth-grade mathematics test: Evidence of construct validity. Storrs, CT: University of Connecticut; 1996.

53. Smith EV. Multiple true-false items: Scoring protocols, reliability, and validity. Storrs, CT: University of Connecticut; 1995.

54. Wang J. Critical values of guessing on true-false and multiple-choice tests. Annual Meeting of the American Educational Research Association; San Francisco, CA; 1995.

55. Graham MT, Isom RM. Bad test… good test: designing classroom tests to accommodate disabled learners. Annual International Convention of the Council for Exceptional Children; Denver, CO; 1994.

56. Halpin G, Halpin G, Arbet S. Effects of number and type of response choices on internal consistency reliability. Percept Mot Skills 1994;79(2):928-30.

57. Pokrajac N, Čulo F. Multiple choice question tests in physiology: a preliminary attempt to apply the minimum pass level. Med Educ 1994;28(5):409-17.

58. Hancock GR, Thiede KW, Sax G, Michael WB. Reliability of comparably written two-option multiple-choice and true-false test items. Educ Psychol Meas 1993;53(3):651-60.

59. Toppino TC, Luipersbeck SM. Generality of the negative suggestion effect in objective tests. J Educ Res 1993;86(6):357-62.

60. Dowd SB. Multiple-Choice and Alternate-Choice Questions: Description and Analysis. 1992.

61. Downing SM. True-false, alternate-choice, and multiple-choice items. Educ Meas 1992;11(3):27-30.

62. Frisbie DA. The multiple true-false item format: A status review. Educ Meas 1992;11(4):21-6.

63. Haladyna TM. The Effectiveness of Several Multiple-Choice Formats. Appl Meas Educ 1992;5(1):73-88.

64. Pinglia RS. A comparative study of true-false, alternate choice, and multiple-choice item formats. Indian J Psychometry Educ 1992;23(1):49-56.

65. Williams JM. Writing Quality Teacher-Made Tests: A Handbook for Teachers. Wheaton, MD; 1992.

66. Van Susteren TJ, Cohen EB, Simpson DE. Alternate-choice test items: implications for measuring clinical judgment. Teach Learn Med 1991;3(1):33-7.

67. Frisbie DA, Becker DF. An analysis of textbook advice about true-false tests. Appl Meas Educ 1990;4(1):67-83.

68. Powell JL, Gillespie C. Assessment: all tests are not created equally. Annual Meeting of the American Reading Forum; Sarasota, FL; 1990.

69. Rubadeau DO, Garrett WA, Rubadeau RJ. Appropriate Testing. Prince George, British Columbia, Canada: College of New Caledonia Press; 1990.

70. Holden C. Big changes urged for precollege Math: new standards would expand content and emphasize concepts and applications over rote learning. Down with true-false tests. Science 1989;243(4899):1655.

71. Kolstad RK, Kolstad RA. Strategies used to answer MC test items by examinees in top and bottom quartiles. Educ Res Quart 1989;13(4):2-5.

72. Norcini JJ, Shea JA, Langdon LO, Hudson LD. First American Board of Internal Medicine critical care examination: process and results. Crit Care Med 1989;17(7):695-8.

73. Petz B. O preferiranju odgovora “točno” u testovima znanja tipa “točno—netočno” [On preference for the answer “true” in achievement tests]. Primijenjena Psihologija 1989;10(1):39-43.

74. Shick J. Tantalizing textbook tests, part II: True-false, matching, completion and essay. Health Educ 1989;20(2):18-23.

75. Toppino TC, Brochin HA. Learning from tests: the case of true-false examinations. J Educ Res 1989;83(2):119-24.

76. Kolstad RK, Creider RD, Kolstad RA. Structural cueing on multiple-choice test items. Education 1987;107(3):315-20.

77. Luecht RM. Test pac: a program for comprehensive item and reliability analysis. Educ Psychol Meas 1987;47(3):623-6.

78. Maihoff NA. A comparison of alternate-choice and true-false item forms used in classroom examinations. East Lansing, MI: Michigan State University; 1987.

79. Smith CW. 100 ways to improve and use teacher-made tests. Illinois School J 1987;66(3):20-6.

80. Cirn JT. True/false versus short answer questions. Coll Teach 1986;34(1):34-7.

81. Frisbie DA, Druva CA. Estimating the reliability of multiple true-false tests. J Educ Meas 1986;23(2):99-105.

82. Van Susteren TJ. The comparative reliability and validity of alternate-choice and multiple-choice tests. East Lansing, MI: Michigan State University; 1986.

83. Vydareny KH, Blane CE, Calhoun JG. Guidelines for writing multiple-choice questions in Radiology courses. Invest Radiol 1986;21(11):871-6.

84. Haladyna TM, Downing SM. A quantitative review of research on multiple-choice item writing. Annual Meeting of the American Educational Research Association; Chicago, IL1985.

85. Kolstad RK, Wagner MJ, Kolstad RA. Format selection in machine-scored classroom achievement tests. J Dent Educ 1985;49(12):799-803.

86. Maihoff NA, Mehrens WA. A comparison of alternate-choice and true-false item forms used in classroom examinations. Annual Researchers Meeting of the National Council on Measurement in Evaluation; Chicago, IL; 1985.

87. Petz B. O preferiranju odgovora “točno” u testovima znanja tipa “točno—netočno” [On preference for the answer “true” in achievement tests]. Revija za Psihologiju 1985;15(1-2):49-54.

88. Varnhagen S, Calder PW. General testing model for microcomputer assessment in education. Annual Meeting of the American Educational Research Association; Chicago, IL, USA1985.

89. Dolinsky D, Reid VE. Types of classroom tests: objective cognitive measures. Am J Pharm Educ 1984;48(3):285-90.

90. Georgi RY. A comparison of two test response formats. Provo, UT: Brigham Young University; 1984.

91. Ory JC. Improving your test questions. Urbana, IL: University of Illinois at Urbana-Champaign; 1983.

92. Barker D, Ebel RL. A comparison of difficulty and discrimination values of selected True-False item types. Contemp Educ Psychol 1982;7(1):35-40.

93. Koeslag JH. Multiple-choice and true-false questions - a test of ignorance? S Afr Med J 1982;61(19):687.

94. McKee BG, Lang HG. A comparison of deaf students’ performance on true-false and multiple-choice items. Am Ann Deaf 1982;127(1):49-54.

95. Weiten W. Relative effectiveness of single and double multiple-choice questions in educational measurement. J Exp Educ 1982;51(1):46-50.

96. Hogan TP. Relationship between free-response and choice-type tests of achievement: a review of the literature. Green Bay, WI: University of Wisconsin-Green Bay; 1981.

97. Koeslag JH, Melzer CW. The incorrect response in multiple-choice examinations. S Afr Med J 1981;60(15):591-2.

98. Ebel RL. The feasibility of using more false than true test items. Acad Psychol Bull 1980;2(1):53-7.

99. Hsu LM. Dependence of the relative difficulty of true-false and grouped true-false tests on the ability levels of the examinees. Educ Psychol Meas 1980;40(4):891-4.

100. Sax G, Reiter PB. Reliability and validity of two-option multiple-choice and comparably written true-false items. Seattle, WA: University of Washington; 1980.

101. Green K. Multiple choice and true-false: reliability and validity compared. J Exp Educ 1979;48(1):42-4.

102. Hsu LM. Ordering power of separate versus grouped true-false tests: interaction of type of test with knowledge levels of examinees. Appl Psych Measurement 1979;3(4):529-36.

103. Ebel RL. The ineffectiveness of multiple true-false test items. Educ Psychol Meas 1978;38(1):37-44.

104. Green K. Multiple choice converted to true-false: comparative reliabilities and validities. Annual Meeting of the Western Psychological Association; San Francisco, CA1978.

105. Knapp TR. The reliability of a dichotomous test-item: A “correlationless” approach. J Educ Meas 1977;14(3):237-52.

106. Larkins AG, Swint JW. Acquiescence-dissent response set on an elementary true-false achievement test. Educ Psychol Meas 1976;36(4):1025-30.

107. Ebel RL. Can teachers write good true-false test items? J Educ Meas 1975;12(1):31-5.

108. Boyd JL. The effect of format change on test item difficulty. Annual Meeting of the Military Testing Association; Oklahoma City, OK1974.

109. Follman J, Hall B, Wiley R, Hartman J. Relationship between objective test formats. Educ Rev 1974;26(2):150-1.

110. Frisbie DA. The effect of item format on reliability and validity: A study of multiple choice and true-false achievement tests. Educ Psychol Meas 1974;34(4):885-92.

111. Olsen HD, Barickowski RS. Test item arrangement and adaptation level. American Educational Research Association Annual Meeting; Chicago, IL; 1974.

112. Sheehan DS, Hambleton RK. A general FORTRAN IV test scoring program. Educ Psychol Meas 1974;34(1):169-71.

113. Ebel RL. Can classroom teachers write good true-false test items? Convention of the National Council on Measurement in Education; Chicago, IL; 1973.

114. Frisbie DA. Multiple choice versus true-false: A comparison of reliabilities and concurrent validities. J Educ Meas 1973;10(4):297-304.

115. Frisbie DA, Ebel RL. Comparative reliabilities and validities of true-false and multiple choice tests. Annual meeting of the American Educational Research Association; Chicago, IL1972.

116. Knight SS. Manipulating response set in the true-false test. East Lansing, MI: Michigan State University; 1972.

117. Lynch DO, Smith BC. To change or not to change item responses when taking. Annual Meeting of the American Educational Research Association; Chicago, IL; 1972.

118. Oosterhof AC, Glasnapp DR. Comparative reliabilities of the multiple choice and true-false formats. Annual Meeting of the American Educational Research Association; Chicago, IL; 1972.

119. Ebel RL. The comparative effectiveness of true-false and multiple choice achievement test items. Annual Meeting of the American Educational Research Association; New York, NY; 1971.

120. Ebel RL. How to write true-false test items. Educ Psychol Meas 1971;31(2):417-26.

121. Frisbie DA. Comparative reliabilities and validities of true-false and multiple choice tests. East Lansing, MI: Michigan State University; 1971.

122. Jackson DN, Neill JA, Bevan AR. An evaluation of forced-choice and true-false item formats in personality assessment. Princeton, NJ: Educational Testing Service; 1971.

123. Ebel RL. The case for true-false test items. School Rev 1970;78:373-89.

124. van der Veen F, Howard KI, Austria AM. Stability and equivalence of scores based on three different response formats. Proceedings of the Annual Convention of the American Psychological Association 1970;5:99-100.

125. van Naerssen RF. Tweekeuze-item in Studietoetsen [Two-choice items in study-tests]. Nederlands Tijdschrift voor de Psychologie 1970;25(6):393-403.

126. Ebel RL. Blind guessing on objective achievement tests. J Educ Meas 1968;5(4):321-5.

127. Avital SM. Comparing combined-response items with single-response items in a Mathematics achievement test. Ont J Educ Res 1967;9(3):175-83.

128. Zern D. Effect of variations in question-phrasing on true-false answers by grade-school children. Psychol Rep 1967;20(2):527-33.

129. Gustav A. Student’s preferences for test format in relation to their test scores. J Psychol 1964;57(1):159-64.

130. Robinson EA. Choice, chance, and cheating. Educ Theor 1957;7:63-8.

131. Carter HD. Importance and significance of objective test items. Calif J Educ Res 1955;6(2):61-71.

132. Travers RMW. How to make achievement tests. New York, NY: The Odyssey Press; 1950.

133. Weimer BR. Some testing procedures in the biological sciences. Turtox News 1949;27:222-8.

134. Wright WAE. The modified true-false item applied to testing in Chemistry. School Sci Math 1944;44(7):637-9.

135. Copeland JS, Gilliland AR. A comparison of the validity and reliability of three types of objective examinations. J Educ Psychol 1943;34(4):242-6.

136. Curtis FD, Darling WC, Henry Sherman N. A study of the relative values of two modifications of the true-false test. J Educ Res 1943;36(7):517-27.

137. Carter HD, Crone AP. The reliability of new-type or objective tests in a normal classroom situation. J Appl Psychol 1940;4(3):353-68.

138. Drake CA. An examination-marking experiment. School Soc 1940;52:95-6.

139. Andrew DM, Bird C. The stability of new-type questions. J Educ Psychol 1938;29(7):501-12.

140. Dunlap JW. The relationship between the type of question and scoring errors. J Exp Educ 1938;6(3):376-9.

141. Bird C, Andrew DM. The comparative validity of new-type questions. J Educ Psychol 1937;28(4):241-58.

142. Buckingham GE, Lee RE. A technique for testing unified concepts in science. J Educ Res 1936;30(1):20-7.

143. Hovland CI, Eberhart JC. A new method of increasing the reliability of the true-false examination. J Educ Psychol 1935;26(5):388-94.

144. Fay PJ, Middleton WC. An economical method of administering and scoring new-type examinations. J Appl Psychol 1934;18(1):77-84.

145. Jordan AM. Objective Tests on Educational Psychology. New York, NY: Holt; 1934.

146. Lee JM, Symonds PM. New type of objective tests: A summary of recent investigations. J Educ Psychol 1934;25(3):161-84.

147. Noll VH. Measuring scientific thinking. Teach Coll Rec 1934;35:685-93.

148. Krueger WCF. Distributions of scores based on correct guessing for true-false test of various lengths. J Educ Psychol 1933;24(3):185-8.

149. Eurich AC. Four types of examinations compared and evaluated. J Educ Psychol 1931;22(4):268-78.

150. Cuff NB. Scoring objective tests. J Educ Psychol 1932;23(9):681-6.

151. Shulson V, Crawford CC. Experimental comparison of true-false and completion tests. J Educ Psychol 1928;19(8):580-3.

152. Fritz MF. Guessing in a true-false test. J Educ Psychol 1927;18(8):558-61.

153. Ruch GM, Stoddard GD. Types and characteristics of objective examinations. Tests and measurements in high school instruction. Yonkers, NY: World Book Company; 1927. p. 266-81.

154. McClusky HY, Curtis FDY. A modified form of the true-false test. J Educ Res 1926;14(3):213-24.

155. Ruch GM, Degraff MH, Gordon WE, MacGregor JB, Maupin N, Murdock JR. Short-Answer examinations in the social studies in the elementary school grades. Public Pers Stud 1926;4(10):274-7.

156. Wood BD. New type examinations in the College of Physicians and Surgeons. J Pers Res 1926;5(6):227-34.

157. Wood BD. New type examinations in the College of Physicians and Surgeons. J Pers Res 1926;5(7):277-83.

158. Remmers HH, Marschat LE, Brown A, Chapman I. An experimental study of the relative difficulty of true-false, multiple-choice, and incomplete-sentence types of examination questions. J Educ Psychol 1923;14(6):367-72.

159. Knight FB. Data on the true-false test as a device for college examination. J Educ Psychol 1922;13(2):75-80.

160. Irvin LK, Halpern AS, Landman JT. Assessment of retarded student achievement with standardized true/false and multiple-choice tests. J Educ Meas 1980;17(1):51-8.

161. Tarnóczy T. Csoportos igaz-hamis testvizsgálatok gyors - és helyes értékelése [A quick and correct evaluation of true-false group tests]. Magyar Pszichológiai Szemle 1938;10:180-6.

162. Dudley A. Multiple dichotomous-scored items in second language testing: Investigating the multiple true-false item type under norm-referenced conditions. Lang Test 2006;23(2):198-228.

163. Sim S-M, Rasiah RI. Relationship between item difficulty and discrimination indices in true/false-type multiple choice questions of a para-clinical multidisciplinary paper. Ann Acad Med Singap 2006;35(2):67-71.

164. Albanese MA, Kent TH, Whitney DR. Cluing in multiple-choice test items with combinations of correct responses. J Med Educ 1979;54(12):948-50.

165. Mayo GD, Siegel AI. A “new” type of true-false item. Psychol Rep 1956;2(3):83-6.
